# Intraoperative Ultrasound in Brain and Spine Surgery: Current Applications, Translational Value and Future Perspectives

**DOI:** 10.3390/neurosci6040113

**Published:** 2025-11-12

**Authors:** Carmelo Pirri, Nina Pirri, Veronica Macchi, Andrea Porzionato, Carla Stecco, Raffaele De Caro

**Affiliations:** 1 Department of Neuroscience, Institute of Human Anatomy, University of Padova, 35121 Padova, Italy; veronica.macchi@unipd.it (V.M.); andrea.porzionato@unipd.it (A.P.); carla.stecco@unipd.it (C.S.); rdecaro@unipd.it (R.D.C.); 2 Department of Medicine—DIMED, School of Radiology, Radiology Institute, University of Padua, 35121 Padova, Italy; nina_92_@hotmail.it

**Keywords:** intraoperative ultrasound, glioma, spinal tumors, cerebrospinal fluid dynamics, contrast-enhanced ultrasound, elastography, neurosurgery, brain shift, spinal decompression

## Abstract

Intraoperative ultrasound (IOUS) has developed from a rudimentary adjunct into a versatile modality that now plays a crucial role in neurosurgery. Offering real-time, radiation-free and repeatable imaging at the surgical site, it provides distinct advantages over intraoperative magnetic resonance (MRI) and computed tomography (CT) in terms of accessibility, workflow integration and cost. The clinical spectrum of IOUS is broad: in cranial surgery it enhances the extent of resection of gliomas and metastases, supports dissection in meningiomas and enables localization of MRI-negative pituitary adenomas; in spinal surgery, it guides resection of intradural and intramedullary tumors, assists in myelotomy planning and confirms decompression in degenerative conditions such as cervical myelopathy and ossification of the posterior longitudinal ligament. IOUS also offers unique insights into cerebrospinal fluid disorders, including arachnoid webs, cysts, syringomyelia and Chiari malformation, where it visualizes cord compression and CSF flow restoration. In trauma and oncological emergencies, it provides immediate confirmation of decompression, directly influencing surgical decisions. Recent innovations, including contrast-enhanced ultrasound, elastography, three-dimensional navigated systems and experimental integration with artificial intelligence and robotics, are extending its functional scope. Despite heterogeneity of evidence and operator dependence, IOUS is steadily transitioning from an adjunctive tool to a cornerstone of multimodal intraoperative imaging, bridging precision, accessibility and innovation in contemporary neurosurgical practice.

## 1. Introduction

Intraoperative imaging has reshaped modern neurosurgery by overcoming the intrinsic limitation of microscopic vision and tactile feedback. Despite advances in neuronavigation and operative microscopes, the ability to reliably determine the extent of lesion removal, to distinguish abnormal from normal tissue and to confirm adequate decompression remains restricted. Among available modalities, intraoperative ultrasound (IOUS) has emerged as one of the most versatile and accessible tools, providing real-time anatomical and functional information directly at the operative site [1,2,3].

Historically, IOUS was introduced in the 1980s for visualizing intramedullary spinal cord lesions, syringomyelia and arachnoid cysts [4,5]. Early probes were hindered by low resolution and artifacts, yet even rudimentary images provided useful intraoperative guidance. Subsequent advances in probe design, image reconstruction and integration with neuronavigation systems addressed these shortcomings and expanded its clinical applications [5,6,7,8].

In neuro-oncology, achieving maximal safe resection is a primary objective, as the extent of resection correlates with survival, recurrence and functional outcomes. Preoperative magnetic resonance imaging (MRI) is indispensable but cannot compensate for intraoperative brain shift or tissue deformation [9]. Here, IOUS has proven especially valuable: systematic reviews and metanalyses confirm that it can detect residual tumors with high specificity and clinically useful sensitivity, thereby increasing the likelihood of gross total resection [10,11]. Compared with intraoperative MRI (iMRI) and intraoperative CT (iCT), IOUS typically allows minute-scale acquisitions and lower capital/logistical requirements [6,12,13]; however, these advantages can be offset by operator dependence, artifact susceptibility, restricted acoustic window and integration/consumable costs. Quantitatively, pooled estimates report residual tumor detection with sensitivity of ~0.75 and specificity of ~0.88 (AUC ~0.89) in glioma cohorts, while comparative series suggest complementary performance versus iMRI (e.g., higher IOUS sensitivity with lower specificity). Navigated 3D-IOUS has been shown to alter intraoperative decisions (prompting additional resection in a notable minority of cases) with strong agreement with postoperative MRI on residual volumes, and a single 3D sweep typically requires ~1–2 min, enabling repeated checks without major workflow penalties, whereas iMRI commonly adds tens of minutes depending on setup [14,15].

Applications extend to spinal surgery, where IOUS facilitates durotomy planning, guides myelotomy and enables real-time confirmation of resection completeness in intramedullary and extramedullary tumors [16,17]. In degenerative conditions such as ossification of the posterior longitudinal ligament (OPLL) and cervical spondylotic myelopathy, IOUS can assess decompression adequacy intraoperatively and may have prognostic value, with intraoperative hyperechogenicity correlating with postoperative outcomes [18].

Emerging innovations, including contrast-enhanced ultrasound, elastography, three-dimensional navigated IOUS and AI-assisted interpretation, show promise in addressing these limitations [8,19,20]. Although still under validation, these advances suggest a future in which IOUS combines accessibility with unprecedented precision, consolidating its role as a core intraoperative imaging modality in neurosurgery. Despite its versatility, IOUS has well-recognized constraints in routine neurosurgical use: image quality and interpretation are operator-dependent; artifacts from air, blood and hemostatic materials can mimic or obscure residual disease; bone/air interfaces restrict the acoustic window and field of view; sensitivity may drop for small or deep-seated lesions; and post-debulking cavity geometry can complicate margin assessment. Heterogeneity in acquisition presets and workflow further limits reproducibility and there is a steep learning curve compared with static imaging. These limitations are partially mitigated by navigated 3D acquisitions and MRI-US coregistration [20,21,22,23,24].

For AI-assisted IOUS (e.g., automated tissue classification, margin detection and registration), deployment must satisfy evolving medical AI regulations. In the European Union, the AI Act classifies clinical AI embedded in devices as high-risk, imposing risk management, data governance, human oversight and post-market monitoring with staged applicability through 2026–2027 [25]. In the United States, the FDA expects Predetermined Change Control Plans to govern iterative model updates within a total product lifecycle approach [26].

Against this backdrop, the present review aims to provide a comprehensive and critical synthesis of the role of IOUS in neurosurgery. Specifically, it will (i) examine evidence supporting its use in cranial and spinal procedures; (ii) compare its strengths and limitations with alternative intraoperative imaging modalities; (iii) identify current gaps in knowledge, in particular with regard to standardization, training and long-term outcomes; and (iv) explore future perspectives, including functional US applications, multimodal integration and the transformative potential of artificial intelligence and robotic systems.

## 2. Materials and Methods

This review was conceived as a narrative synthesis of the literature on IOUS in neurosurgery, with the goal of integrating technical, clinical and practical perspectives. A structured search of the literature was conducted in the PubMed, Scopus and Web of Science databases for studies published between January 1982 and September 2025. The following search terms and combinations were used: (“intraoperative ultrasound” OR “IOUS”) AND (“neurosurgery” OR “brain tumor” OR “glioma” OR “spine” OR “meningioma” OR “Chiari” OR “myelopathy” OR “intramedullary tumor”). Original studies, reviews and technical notes describing the application, accuracy and clinical impact of IOPUS in cranial or spinal surgery were included. Case reports were included only when they provided unique technical insights. Articles not in English or unrelated to human neurosurgery were excluded.

## 3. Historical Context and Technological Evolution

**Figure 1:**
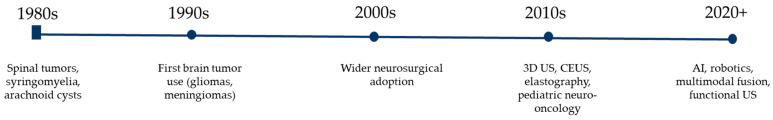
Evolution and applications of iOUS in neurosurgery. Timeline illustrating the progressive adoption of IOUS from its introduction in the 1980s for intramedullary lesions and CSF pathologies, to contemporary applications in brain and spine oncology and degenerative and traumatic conditions and the integration of advanced modalities such as 3D navigation, CEUS, elastography, AI and robotics.

The adoption of IOUS in neurosurgery dates back to the late 1970s and early 1980s. initial reports by Dohrmann and Rubin [4,5], and later Jokich et al. [6], demonstrated its feasibility in visualizing syringomyelia, arachnoid cysts and intramedullary spinal cord tumors [4,5,6]. At that time, images were rudimentary, with low resolution and significant artifacts, yet they provided crucial intraoperative feedback not otherwise attainable. These studies proved that real-time imaging could extend the surgeon’s perception beyond the microscope and tactile exploration, offering a dynamic and functional assessment of neural structures [4,5,6]. Technological advances subsequently reshaped the field. High-frequency transducers improved resolution, advanced beamforming algorithms reduced artifacts and volumetric imaging allowed reconstruction of complex geometries. The integration of US in neuronavigational workflows was transformative, mitigating registration errors due to intraoperative brain shift [1,7] (Figure 1).

Unlike intraoperative MRI (iMRI) or computed tomography (iCT), which require costly infrastructure and prolong operative time, IOUS emerged as a rapid, radiation-free and repeatable modality. Nevertheless, practical constraints can limit sustained adoption in resource-limited settings, such as procurement and maintenance of US machines and probes, availability and cost of sterile probe covers (and, when used, CEUS agents), reliable service/sterilization and power and constrained training and reimbursement pathways, which also affect reproducibility across teams [27]. Beyond these logistical aspects, IOUS has intrinsic imaging limitations; its lower soft tissue contrast resolution compared with MRI can make a subtle differentiation between tumor, edema and normal parenchyma more challenging; depth penetration and field of view are restricted by bone and air interfaces; and acoustic shadowing or reverberation artifacts may obscure deep margins [27,28]. Interpretation challenges further arise from IOUS’s lower soft tissue contrast relative to MRI, restricted acoustic windows near bone/air and susceptibility to artifacts from air, blood and hemostatic materials; together with platform/preset variability and non-uniform techniques (e.g., saline fill and pressure control), these factors can reduce sensitivity for small or deep-seated remnants and hinder external validation [28]. That said, case studies from low- and middle-income settings document the feasibility and workflow impact of IOUS when paired with basic training, checklists and systematic archiving/QA, supporting its role as an accessible adjunct while acknowledging the above constraints [29,30]. Its affordability and portability position it as a democratizing force in intraoperative imaging, with applications not only in high-resource academic centers but also in low- and middle-income countries where iMRI is not feasible [1,7] (Table 1).

## 4. Brain Tumor Surgery: Scope, Innovation and Clinical Outcomes

The application of IOUS in brain tumor surgery has evolved from a supplementary imaging modality into an integral component of contemporary neuro-oncology practice. Among its most studied indications are gliomas, in which maximizing the extent of resection (EOR) directly influences patient survival, recurrence and functional outcomes. Static preoperative MRI, although invaluable for planning, cannot compensate for intraoperative anatomical changes, including brain shift, tissue swelling and cavity deformation [1,9,10,11]. Numerous systematic reviews and meta-analyses have demonstrated that iOUS reliably detects residual tumor with high specificity and clinically meaningful sensitivity, findings that align closely with postoperative MRI [31,32,33]. Larger multicenter series have further confirmed that navigated IOUS enhances resection control and has been associated with improved survival metrics in some cohorts; however, the available evidence is predominantly retrospective and heterogeneous, so causal inference is not warranted [34].

The versatility of IOUS is underscored by its adaptability to a range of tumor types. In gliomas, conventional B-mode remains the cornerstone for intraoperative margin assessment, but advanced modalities such as contrast-enhanced ultrasound (CEUS) and elastography are progressively redefining its potential [21,31]. CEUS allows the surgeon to appreciate vascular heterogeneity within tumors, enhancing delineation of residual tissue, especially in high-grade gliomas [34,35,36]. Elastography, on the other hand, quantifies tissue stiffness, offering a biomechanical contrast between infiltrating tumors, edematous brain and necrotic tissue [37,38]. Navigated three-dimensional (3D) ultrasound further integrates volumetric data with preoperative MRI, enabling quantitative correction for brain shift and dynamic reoptimization of the surgical plan [39,40].

Beyond gliomas, IOUS has proven its worth in other cranial neoplasms. In metastatic brain tumors, intraoperative imaging with US significantly increases gross total resection (GTR) rates, a factor of particular importance in patients with large or multiple lesions [41,42]. Similarly, CEUS has been employed in meningiomas to visualize tumor vascularization, improve dissection planes and anticipate intraoperative bleeding risks [43]. Pediatric neuro-oncology represents another domain in which IOUS is particularly useful. In children, where small craniotomies, deep-seated lesions and the need to minimize resection-related morbidity pose unique challenges, navigated IOUS shows high diagnostic performance for residual detection (e.g., sensitivity 100% and specificity 84.6%) and strong agreement with iMRI for volume assessment and spatial accuracy [7]. Series focused on pediatric brain tumors also reported very high concordance with postoperative MRI in determining EOR (e.g., NPV 98% and PPV 100% for GTR/subtotal calls) [44]. Moreover, the largest pediatric series to date from a tertiary children’s center confirms high sensitivity/specificity of IOUS in predicting residual disease and highlights its value for intraoperative decision-making [45]. Multicenter experience in pediatric low-grade glioma further supports the complementary roles of IOUS and iMRI in maximizing safe resection. Vascular lesions such as cavernomas and hemangioblastomas also benefit from IOUS, as it allows intraoperative identification of lesion margins and feeding vessels. Furthermore, reports describe its utility in guiding aspiration of abscesses, offering immediate feedback on cavity decompression [46,47,48].

## 5. Pituitary and Sellar Pathology

Surgery in the sellar and parasellar region is inherently challenging because of the small dimensions of the pituitary gland, the frequent presence of microlesions and the close relationship with the cavernous carotids and optic chiasm. This is evident in Cushing disease, where corticotroph microadenomas are often <5 mm and remain undetectable even on dedicated MRI sequences [49]. IOUS has therefore gained attention as a complementary imaging modality capable of revealing adenomas that are radiologically occult. Various reports have shown that IOUS through the trans-sphenoidal corridor can delineate hypoechoic adenomatous tissue guiding selective adenomectomy and may be associated with higher biochemical remission rates in selected series [50,51]. In a comparative series of 138 trans-sphenoidal surgeries, the use of IOUS was associated with higher GTR (79% vs. 44%; *p* = 0.0008), shorter operative time (74 vs. 146 min; *p* < 0.0001), lower estimated blood loss (119 vs. 284 mL; *p* < 0.0001) and reduced length of stay (2.9 vs. 4.2 days; *p* = 0.001); overall complication rates were not increased (numerically lower, without statistical significance) [51]. These data support the safety of IOUS-assisted trans-sphenoidal surgery while underscoring that the evidence remains observational [52,53]. Given the observational nature and variability of criteria across studies, these findings should be interpreted cautiously and not as proof of causality [50,51]. Moreover, IOUS provides real-time confirmation of tumor removal and helps distinguish residual adenoma from normal pituitary, avoiding unnecessary exploration. Doppler-enabled US adds another layer of safety, enabling mapping of sellar and parasellar vessels, in particular being valuable in reoperations or invasive macroadenomas where surgical planes are obscured [54]. Beyond adenomas, IOUS has also been explored in cystic sellar lesions (e.g., Rathke’s cleft cysts and craniopharyngiomas), where it supports cyst aspiration and assessment of residual cavity [55].

## 6. Spinal Pathology: Tumors, Degenerative Disorders and Deformity

IOUS has long been established as a reliable adjunct in spinal surgery, with applications spanning intradural tumors, intramedullary lesions, degenerative disorders and deformity correction. In intradural–extramedullary tumors, such as meningiomas and schwannomas, IOUS allows surgeons to localize the lesion before durotomy, refine the dural opening and confirm adequate decompression following resection [56,57]. Particularly illustrative are “mobile” schwannomas, which can migrate within the dural sac and present at different levels intraoperatively compared to preoperative MRI. In such cases, IOUS is indispensable for accurate localization and complete exposure, preventing unnecessary durotomy or incomplete resection [57]. In intramedullary tumors, including ependymomas, astrocytomas, gangliogliomas and hemangioblastomas, the role of IOUS is equally significant. US enables the delineation of the cord–tumor interface, facilitates the choice of a safe myelotomy corridor and assists in identifying cleavage planes between pathological and normal tissue [58]. IOUS defined tumor margins and reduced the risk of neurological deficits following resection of intramedullary ependymomas [59,60]. More recent developments, such as navigated three-dimensional (3D) IOUS, have expanded these capabilities, offering real-time volumetric guidance that reduces navigation error and increases surgical confidence [61]. This technology has also proven effective in the management of intramedullary vascular lesions, such as hemangioblastomas and dural arteriovenous fistulas, where intraoperative mapping of feeding vessels is critical for safe dissection and complete obliteration [62]. Beyond oncological indications, IOUS is gaining relevance in degenerative and deformity surgery. In degenerative cervical myelopathy, IOUS markers provide measurable prognostic information. In a prospective cohort, the mean 12-month modified Japanese Orthopaedic Association score recovery rate was 68.6 ± 20.3% overall; patients with adequate sonographic cord re-expansion after decompression achieved 76.2 ± 16.2% versus 59.2 ± 21.7% in those with inadequate re-expansion (*p* = 0.028). Hyperechogenicity was more frequent in the latter group, indicating that real-time IOUS expansion correlates with better neurological recovery [63]. Complementarily, intraoperative cord hyperechogenicty intensity (gray-value ratio) showed a negative correlation in 12-month recovery (ρ = −0.582, *p* = 0.0049) and sensory (ρ = −0.452, *p* = 0.035) subscores, reinforcing its prognostic value; mean recovery in that series was 65 ± 20.3% [64]. Contrast-enhanced IOUS perfusion offers complementary insight: in a prospective study (n = 26), the overall recovery rate was 50.7 ± 33.3%; peak intensity (PI) was higher in compressed vs. normal cord (24.58 ± 3.19 vs. 22.43 ± 2.39; *p* = 0.019); and both ΔPI and ΔAUC correlated negatively with recovery (r = 0.463, *p* = 0.030; r = −0.466, *p* = 0.029), with worse outcomes in hyperechoic cords (*p* = 0.016) [65]. For intramedullary spinal cord tumors, IOUS guidance enhances resection control and functional outcomes. In a contemporary single-institution series of 43 patients, IOUS confirmed lesion extent and location before dural opening in 97.7%, detected residual or hidden lesions in 7%, verified absence of hematoma in 53.5% and avoided additional durotomies in 7%; GTR was achieved in 93%, supporting both oncologic and neurological benefits [66]. For ossification of the OPLL, IOUS has been employed both in anterior and posterior decompression, confirming the adequacy of spinal canal enlargement and reducing the risk of under-decompression [18]. Similarly, in technically demanding procedures such as oblique corpectomy, IOUS offers additional safety by identifying the course of the vertebral artery and assessing the adequacy of ventral decompression, minimizing the likelihood of vascular complications [49]. In laminoplasty, the modality provides dynamic intraoperative confirmation of canal expansion and restoration of spinal cord pulsatility, which has been reported to correlate with postoperative neurological improvement in observational series [50,67]. These correlations do not establish causality. Finally, in the domain of complex spinal deformity surgery, IOUS has proven to be highly adaptable. During pedicle subtraction osteotomy (PSO), real-time sonographic assessment of spinal cord morphology before and after correction mitigates the risk of acute neurological deterioration, as reported by Chryssikos [68]. In scoliosis correction, IOUS has been successfully used to assess spinal canal dimensions intraoperatively and to monitor dynamic changes in cord alignment, offering an additional safeguard beyond neurophysiological monitoring. Taken together, these applications confirm that IOUS is not limited to tumor surgery but has evolved into a multipurpose modality, capable of guiding diverse spinal interventions with direct implications for both surgical precision and patient safety.

## 7. Cerebrospinal Fluid Dynamics and Arachnoid Pathologies

One of the most distinctive contributions of IOUS lies in the assessment of cerebrospinal fluid (CSF) dynamics and arachnoid pathologies, a domain in which conventional MRI often underperforms due to limitations in resolution and inability to capture real-time CSF flow. Lesions such as arachnoid webs and cysts frequently evade preoperative imaging, yet IOUS offers direct, dynamic visualization of these abnormalities and their effects on spinal cord morphology. Castillo et al. [69] demonstrated that IOUS could confirm the presence of dorsal arachnoid webs and provide real-time evidence of restored CSF pulsatility after surgical fenestration, thus serving as both a diagnostic and monitoring tool during surgery [69]. Similarly, Takahata et al. [70] reported the identification of pulsating arachnoid cysts as hidden etiologies of progressive thoracic myelopathy, visualized intraoperatively by IOUS when preoperative MRI was inconclusive [70]. The role of IOUS in syringomyelia is well established and historically among the earliest spinal applications of this modality. Dohrmann and Rubin [4,5] first reported in the 1980s the ability of IOUS to delineate syrinx cavities and to monitor their collapse following decompression [4,5]. Subsequent studies have confirmed that IOUS remains a reliable intraoperative method for guiding syrinx fenestration or syringo-subarachnoid shunting, offering the surgeon dynamic feedback on cavity decompression and CSF flow restoration [5]. In Chiari I malformation, IOUS has been increasingly employed to evaluate intraoperative adequacy of decompression. By combining B-mode imaging with Doppler, surgeons can directly assess CSF flow across the foramen magnum before and after bony decompression or duraplasty [71,72]. Fan et al. [73] showed that IOUS effectively differentiates patients who require only suboccipital craniectomy form those in whom duraplasty or arachnoid lysis is warranted, thereby optimizing the surgical strategy and reducing the risks of under- or overtreatment [73]. However, some authors have cautioned that intraoperative flow measurements may be influenced by patient positioning and anesthetic conditions, highlighting the importance of correlating IOUS findings with preoperative cine-MRIO studies [74,75,76,77]. Furthermore, IOUS has proven useful in inflammatory arachnoidopathies, such as arachnoiditis following epidural anesthesia or prior to surgery. Sklar et al. [78] described how IOUS delineated adhesive arachnoid changes and confirmed cord tethering, findings that were not readily apparent on MRI [78]. In such cases, iOUS contributes both to diagnosis and to guiding targeted lysis of adhesions, with reports describing postoperative functional improvement; nonetheless, attribution of benefit directly to IOUS guidance is not demonstrable given the study designs. Taken together, these applications underscore the unique functional value of IOUS in disorders of CSF circulation and arachnoid pathology. Formal learning curve data specific to CSF and arachnoid pathology are sparse, but technical series and feasibility studies indicate that proficiency depends on the following: (1) differentiating membranes/webs from flow artifacts related to respiration, cardiac pulsatility, probe pressure and positioning; (2) routine use of cine color–Doppler loops to confirm restoration of CSF pulsatility after each surgical step; and (3) correlation with postoperative MRI/cine-MRI to calibrate interpretation [72,73]. In practice, early experience of ~10–15 supervised cases (Chiari I, arachnoid webs/cysts, syrinx) combined with systematic video review and standardized acquisition (stable insonation angle, minimal probe pressure, coordination with ventilation holds) appears to flatten the learning curve and reduce false positives from reverberation or shadowing [79]. These points are supported by the IOUS series in Chiari I (monitoring CSF flow across the foramen magnum and guiding duraplasty decisions), arachnoid web/cysts (localization and confirmation of decompression) and syringomyelia (guiding fenestration/shunt placement) [72,73,74,75,76,77,78,79]. By providing real-time confirmation of lesion presence, cord compression and restoration of CSF flow after intervention, IOUS fills a critical gap left by static imaging modalities.

## 8. Trauma, Infection and Urgent Scenarios

The value of IOUS becomes particularly evident in urgent and emergent neurosurgical scenarios, where timely and accurate intraoperative decision-making is critical and advanced modalities such as iMRI are rarely feasible. In acute cervical spinal cord injury (SCI), early and adequate decompression is one of the most important predictors of neurological recovery. Real-time IOUS offers immediate verification of decompression adequacy, providing dynamic visualization of residual cord compression and subarachnoid space re-expansion. Chryssikos et al. [76] demonstrated that IOUS correlated strongly with postoperative MRI and CT myelography, and in several cases directly influenced intraoperative decision-making by prompting surgeons to extend decompression before closure [76]. This real-time feedback may improve the adequacy of decompression, but evidence from randomized studies is lacking. In metastatic spinal cord compression, where “separation surgery” aims to achieve circumferential decompression of the cord and create a safe margin for subsequent stereotactic radiosurgery, IOUS has proven particularly useful. Ramirez Ferrer et al. [77] showed that IOUS confirmed ventral clearance and cord re-expansion, ensuring that decompression goals were achieved without the morbidity of excessive bony resection [77]. The ability to visualize real-time cord dynamics intraoperatively reduces reliance on postoperative imaging and increases confidence in surgical adequacy, which may translate into more effective combined surgery–radiotherapy workflows; however, causal effects on oncological outcomes have not been established. The utility of IOUS extends to infectious pathologies of the spine. In pediatric cases of spinal epidural abscess and spondylodiscitis, IOUS has been used intraoperatively to confirm adequate evacuation of purulent material and decompression of the neural elements [79]. Given the frequent urgency of such cases, and the challenge of obtaining iMRI in acutely ill children, IOUS provides a safe, rapid and repeatable imaging option. Similarly, in adult epidural abscesses, IOUS may assist in delineating the extension of the collection and ensuring thorough decompression. In trauma-related pathologies beyond SCI, IOUS has been applied to cases involving retropulsed bone fragments, burst fractures and spinal epidural hematomas. Here, IOUS provides real-time reassurance that the canal has been adequately decompressed after laminectomy or corpectomy. Reports highlight its value in confirming ventral decompression when direct visualization is limited by residual vertebral body or posterior longitudinal ligament [80,81]. In emergency surgery, several factors can compromise IOUS image quality and interpretation. Hemodynamic instability and low perfusion pressure may reduce echogenic contrast between neural and surrounding tissues, particularly in acute spinal cord injury or infection, where edema and congestion obscure normal interfaces. Limited acoustic windows due to bone fragments, dural hematoma or constrained exposures restrict insonation angles and depth penetration [77,78]. The presence of blood, air or debris within the operative field introduces reverberation artifacts that can mimic residual compression, while continuous suction or irrigation during decompression can degrade resolution. Patient positioning and ventilatory motion further influence Doppler or dynamic assessments. To mitigate these challenges, optimized techniques include the following: (1) maintaining a stable acoustic window with warmed, de-gassed saline and minimal probe pressure; (2) using low to moderate gain and tissue harmonic imaging to enhance contrast; (3) performing repeat sweeps after hemostasis and irrigation to clear artifacts; and (4) integrating IOUS findings with clinical and neurophysiological monitoring when signal quality is suboptimal [76,77,78,79,80,81,82]. Although IOUS remains feasible under urgent conditions, these constraints underscore the need for operator experience and structured training in emergency workflows. Moreover, the ability to rapidly repeat the examination after corrective maneuvers or hemostasis allows the surgeon to adapt the procedure without delay (Table 2).

## 9. Minimally Invasive and Endoscopic Applications

The progressive miniaturization of surgical approaches has reshaped both spinal and cranial neurosurgery, emphasizing the need for intraoperative imaging modalities that can provide precise guidance without increasing procedural morbidity. In this context, IOUS has emerged as a particularly attractive adjunct, offering portability, repeatability and real-time feedback within narrow operative corridors [28,82]. Compared with iMRI or iCT, IOUS reduces logistical demands and eliminates radiation exposure while maintaining the ability to confirm key anatomical landmarks. Its extension into minimally invasive and endoscopic surgery reflects a natural evolution of these advantages. Applications in full-endoscopic spinal surgery demonstrate feasibility for level localization, endoscope docking and decompression confirmation, thereby significantly decreasing dependence on fluoroscopy [83]. Similarly, in extradural decompression for lumbar stenosis and disc herniation, IOUS has shown strong concordance with postoperative MRI in evaluating adequacy, providing the opportunity to detect residual compression intraoperatively [84]. In cranial neuroendoscopy, IOUS introduced through the working sheath allows for more accurate ventricular access, facilitates colloid cyst resection and supports intraventricular tumor biopsies, in particular in patients with distorted ventricular anatomy [85,86,87]. Collectively, these developments highlight the growing relevance of iOUS as a complementary modality that enhances safety and precision in minimally invasive neurosurgical paradigms.

## 10. Practical IOUS Techniques: Pearls, Pitfalls and Checklists

Effective IOUS hinges on appropriate probe selection, proactive artifact control and timing of acquisitions. For cranial work, a micro-convex 3–8 MHz transducer (global activity mapping, deeper planes) paired with a linear 7–12 MHz probe (cortical margins and superficial residuals) covers most needs; in narrow corridors or pediatrics, a “hockey-stick” 10–15 MHz probe offers excellent near-field resolution and maneuverability. In spinal cases, a linear 7–15 MHz probe through the dural window is preferred for defining the cord–tumor interface and polar vessels; when access is constrained or a deeper field is required, a micro-convex 5–8 MHz is useful. Initial presets should emphasize an adequate depth (then narrowed), focal zone at the structure of interest (or 5–10 mm deeper), dynamic range of ~60–80 dB and tissue harmonics when available, while respecting the ALARA (lowest output consistent with diagnostic quality) [18,19,20,21,22,23,24,25,26,27,28,29,30,31,32,33,34,35,36,37,38,39,40,41,42,43,44,45,46,47,48,49,50,51,52,53,54,55,56,57,58,59,60,61,62,63,64,65,66,67,68,69,70,71,72,73,74,75,76,77,78,79,80,81,82]. Doppler and, where applicable, CEUS can complement vascular mapping. The saline-fill technique is central in cranial resections: use warm, de-gassed saline (~37 °C); overfill the cavity slightly and “burp” slowly to evacuate microbubbles. Keep the transducer fully submerged with minimal pressure to maintain a continuous fluid column; avoid air trapping under cavity edges or bridging veins. Hemostatic agents should be removed or temporarily displaced before the final sweep, as they can appear as hyperechoic mats with posterior shadowing and mimic residual tumors. Never use gel intracavitarily. Common artifacts have recognizable patterns and straightforward remedies [18,19,20,21,22,23,24,25,26,27,28,29,30,31,32,33,34,35,36,37,38,39,40,41,42,43,44,45,46,47,48,49,50,51,52,53,54,55,56,57,58,59,60,61,62,63,64,65,66,67,68,69,70,71,72,73,74,75,76,77,78,79,80,81,82]. Air/reverberation/comet tail produce shimmering hyperechoic lines and dropout, and should be treated with irrigation using de-gassed saline, a slight probe tilt and refilling corners. Blood/clot/turbulence yield diffuse echogenicity or flicker, and should be treated using gentle suction and irrigation, pausing a few seconds for flow to settle and reducing overall gain. Bone edges/kerfs cause acoustic shadowing, and should be treated by changing the acoustic window or angle [18,19,20,21,22,23,24,25,26,27,28,29,30,31,32,33,34,35,36,37,38,39,40,41,42,43,44,45,46,47,48,49,50,51,52,53,54,55,56,57,58,59,60,61,62,63,64,65,66,67,68,69,70,71,72,73,74,75,76,77,78,79,80,81,82]. After duraplasty or patch placement, expect interface echoes and acquire references images before closure to aid interpretation. Sweep timing should be protocolized. For gliomas, obtain (1) a baseline sweep after dural opening to establish orientation and the brain shift baseline; (2) a mid-resection sweep at ~50–70% estimated EOR to redirect the trajectory; and (3) a final sweep after hemostasis and saline refill, before definitive hemostatic placement/closure. For intramedullary tumors, (1) pre-myelotomy scanning identifies the safe corridor (median sulcus vs. dorsal root entry zone (DREZ)) and polar/feeding vessels; (2) after internal debulking, reassess the tumor–cord plane and search for satellite nodules; and (3) before dural closure, confirm cord re-expansion and pulsatility. For Chiari/CSF disorders, (1) obtain a baseline assessment of qualitative CSF flow and tonsillar impaction; (2) reassess after bony decompression to decide on duraplasty/arachnoid lysis; and (3) confirm restoration of CSF pulsatility after duraplasty/lysis. If turbulence or microbubbles persist, it is prudent to wait 2–3 min and repeat the acquisition (Table 3) [18,19,20,21,22,23,24,25,26,27,28,29,30,31,32,33,34,35,36,37,38,39,40,41,42,43,44,45,46,47,48,49,50,51,52,53,54,55,56,57,58,59,60,61,62,63,64,65,66,67,68,69,70,71,72,73,74,75,76,77,78,79,80,81,82].

Typical post-debulking pitfalls include cavity collapse with hidden recesses (mitigate with 360° probe rotation and deliberate oblique sweeps), re-introduction of air due to saline egress (maintain slight overfill and low, peripheral suction) and hemostatic agent mimicry (scan before placement or temporarily remove and re-scan). Reoptimize gain and focus whenever the effective imaging depth changes. When navigation diverges due to brain shift, perform 3D ultrasound sweep (if available) to update the dataset, or when that threat is absent, use IOUS as the real-time reference for margin assessment. To address the learning curve, a pragmatic training pathway can be anchored to the structure and outcomes of the international IOUS course [88], which has been delivered 21 times across 12 countries in a 1–2-day format combining approximatively 4 h of didactics with ~4 h of supervised hands-on (4–7 scanners; ≤5 learners per device) and smartphone-based Neurostream simulation on patient-specific 3D IOUS datasets coregistered to preoperative MRI. In four recent editions (Cape Town 12/2023; Edinburgh 01/2024; Barcelona 04/2024), matched pre/post-surveys (n = 67; 100% response) demonstrated significant improvements: median familiarity increased from 4 (IQR 2–6) to 8 (7–8); comfort rose for machine functionality from 4 (2–5.5) to 7.5 (6–8.5), probe selection from 4 (2–6) to 8 (6.5–9), image acquisition from 4 (3–6) to 7.5 (7–8.5) and interpretation from 4 (3–6) to 7.5 (7–8.5) (all *p* < 0.0001). Procedure-specific comfort also improved for ventricular catheter insertion from 3 (1.5–5) to 7 (6.5–8), endoscopic procedures from 2.5 (1.5–4) to 7 (5–8), tumor resection from 3.5 (2–5.5) to 7 (6–9) and Chiari I decompression from 2 (1.5–4) to 5.5 (4–7) (all *p* < 0.0001); the likelihood of future IOUS use rose to a median of 9/10 [88]. In stratified analyses, post-course scores favored qualified neurosurgeons for probe selection (*p* = 0.03), ventricular catheter insertion (*p* = 0.02) and endoscopic procedures (*p* = 0.029). Reported limitations include self-selected cohort and simulators emphasizing B-mode with limited Doppler/CEUS/elastography integration, underscoring the need to assess knowledge retention and real-world adoption after training [88].

## 11. Discussion

Over the past decades, IOUS has evolved from a complementary adjunct into a versatile imaging modality that continues to broaden its indications in neurosurgery. The field is now witnessing a convergence of three parallel trajectories: the incorporation of iOUS into minimally invasive and endoscopic procedures, the development of novel ultrasound-based functional and intelligent technologies and the integration of IOUS into multimodal intraoperative ecosystems. These advances hold promise to reshape neurosurgical workflows, yet they also highlight persistent limitations and the urgent need for standardized validation. While early generations of IOUS were predominantly anatomical, recent innovations have expanded its scope into functional, biomechanical and even intelligent domains. CEUS has emerged as a valuable adjunct for brain tumor surgery, offering superior delineation of tumor margins and perfusion characteristics compared with conventional B-mode imaging. Prospective comparative studies report that CEUS facilitates differentiation between tumor and peritumoral edema and improves detection of residual tumor tissue [89]. These studies are mostly nonrandomized; thus, statements of benefit should be considered associational. Shear-wave elastography (SWE) further extends this paradigm by quantifying tissue stiffness. Given that gliomas and metastases often differ in their biomechanical properties from surrounding parenchyma, SWE has been investigated as an intraoperative tool for grading and for guiding resection margins. Meanwhile, microflow or superb microvascular imaging (MFI/SMI) provide visualization of the microvasculature beyond the resolution of Doppler techniques, potentially guiding resection in highly vascular tumors. These functional modalities illustrate a broader transition from US as a purely structural modality toward a multiparametric intraoperative imaging platform. In parallel, the incorporation of artificial intelligence (AI) into IOUS interpretation represents one of the most transformative directions. Deep learning algorithms have been trained to automate tissue classification, reduce operator-dependent artifacts and even detect residual tumor in near real time. Experimental systems for spine surgery demonstrate accurate recognition of neural structures during decompression [19], while multicenter initiatives such as BraTioUS have validated deep learning-based glioma segmentation on IOUS across heterogeneous datasets [90]. These developments underscore that AI has the potential not only to standardize interpretation but also to democratize access to expertise in centers where specialized neuroradiological support is not readily available. Another frontier lies in robotic probe manipulation. Standardized 3D sweeps are autonomously performed by robotic frameworks, aiming to reduce operator variability and generate reproducible volumetric datasets. Proof of concept studies demonstrate feasibility of autonomous probe positioning in neurosurgical settings [91]. By ensuring consistent image acquisition, robotic systems could enhance reproducibility and facilitate the incorporation of IOUS datasets into neuronavigation platforms. Finally, the steep learning curve of IOUS, long recognized as a barrier to widespread adoption, is now being addressed by simulation platforms such as USim. These tools allow trainees to rehearse case-specific scenarios, practice probe handling and correlate sonographic findings with known anatomical models [92]. Such training innovations are crucial for integrating IOUS into standard curricula and for reducing inter-operator variability, in particular in centers where exposure to high case volumes is limited. The trajectory of IOUS is increasingly defined not by its role as a stand-alone modality, but as a part of multimodal intraoperative ecosystems. The rationale is compelling: each imaging modality contributes unique strengths but also suffers intrinsic limitations. IOUS offers real-time flexibility and repeatability, while iMRI provides comprehensive anatomical reference at the cost of high resource demands. Fluorescence-guided surgery with 5-ALA or fluorescein visualizes tumor infiltration at the cortical surface but may underestimate deeper components. Neurophysiological monitoring maps functional pathways but lacks spatial anatomical correlation. When combined, these techniques create a more comprehensive framework for surgical decision-making. Multimodal protocols integrating neuronavigation, IOUS, 5-ALA fluorescence and iCT or PET have been associated with an increased extent of resection and lower complication rates in nonrandomized studies [42,93,94]. Given potential confounding and selection bias, causal improvements cannot be inferred. These benefits extend beyond oncology: recent evidence indicates that multimodal imaging, including IOUS, also correlates with favorable intraoperative decision-making and perioperative metrics in reconstructive and complex cranial base procedures [95]. Long-term outcome effects remain to be defined. Such findings suggest that IOUS may function as the “dynamic element” within a multimodal ecosystem, providing continuous intraoperative updates that anchor static modalities like neuronavigation or preoperative imaging.

**Figure 2:**
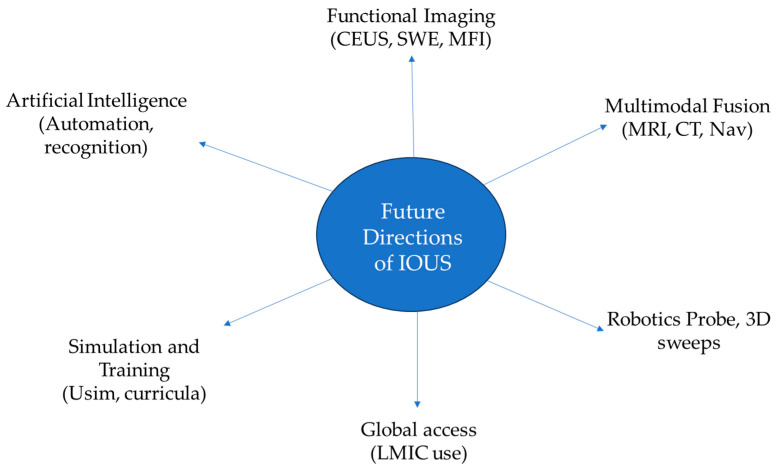
Future directions of iOUS: transition of IOUS from a supportive adjunct to a core component of multimodal intraoperative imaging.

Despite significant progress, the current evidence base for IOUS remains limited and heterogenous. The majority of available studies are retrospective, single-center series with small sample sizes [28,82]. Acquisition protocols vary widely between centers, and interpretation remains strongly operator-dependent, undermining reproducibility and external validity. Although short-term outcomes such as extent of resection or decompression adequacy are frequently reported, data linking IOUS use to survival, recurrence or durable neurological function are insufficient to support causal claims at this time. Future directions must prioritize standardization and validation. Development of consensus-based acquisition protocols and structured reporting guidelines would be instrumental in facilitating reproducibility across centers. Multicenter prospective studies with predefined outcome measures are urgently required to validate the oncological and functional benefits of IOUS. These efforts could mirror the trajectory of fluorescence-guided surgery, where standardization of dosing, lighting and interpretation protocols was critical to widespread adoption. Another important frontier is the integration of emerging functional modalities and AI-driven interpretation into routine workflow. Global equity also represents an underappreciated dimension of IOUS. Reports from low- and middle-income countries demonstrate the feasibility of IOUS implementation in resource-limited settings, with meaningful improvements in safety and surgical precision [41] (Figure 2).

## 12. Conclusions

Intraoperative US is emerging as a key adjunct in neurosurgery; its trajectory is defined by an expanding spectrum of applications, from minimally invasive surgery to neuroendoscopic interventions and from functional tumor imaging to robotic-assisted acquisition. Yet its transformative potential lies in its integration with intelligent algorithms, with multimodal imaging and with global health strategies. Realizing this potential will require prospective, preferably multicenter studies that report effect sizes for survival and functional endpoints and are designed to mitigate bias, alongside standardized protocols and equitable dissemination. Only then will IOUS fully mature from a versatile adjunct into a cornerstone of neurosurgical practice.

## Figures and Tables

**Table 1:** Comparative table: IOUS vs. iMRI vs. iCT vs. fluorescence/IONM.

Feature/Modality	IOUS	Intraoperative MRI	Intraoperative CT	Fluorescence (5-ALA/ICG)	IONM
Typical indications	Brain and spine tumors, Chiari, syringomyelia, decompression check	Glioma, pituitary, deep lesions	Skull base, spine instrumentation	Glioma resection margins, vascular flow	Functional pathway preservation
Imaging type	Real-time, radiation-free, portable	High-field MRI	X-ray-based	Optical contrast	Electrophysiological
Workflow/logistics	Integrated in standard OR, low cost, minimal setup	Requires dedicated suite; high cost, longer setup	Moderate cost; requires shielding	Simple setup; minimal time	Integrated; depends on expertise
Artifact sources	Air, blood, hemostatic materials, bone reflection	Susceptibility, motion	Metal artifacts	Background fluorescence	Electrical noise
Repeatability	Unlimited, real-time	Limited by logistics	Limited by radiation	Limited by dye kinetics	Continuous
Training needs	Moderate; learning curve of ~20–30 cases	High; radiology support	Moderate	Low–moderate	High; subspecialty training
Main advantages	Real-time feedback, cost-effective, broad applicability	Highest soft tissue contrast	Bone visualization, navigation	Tumor margin enhancement	Functional protection
Main limitations	Operator-dependent, artifacts after debulking	Cost, time, accessibility	Radiation, limited soft tissue contrast	Specific to fluorophore uptake	No anatomical visualization

**Table 2:** Neurosurgical spectrum of IOUS in brain and spinal surgery.

Pathology	Role of IOUS	Benefits	Limitations	Level of Evidence
Gliomas [31,32,33]	Residual detection, brain shift correction	↑ GTR, real-time updates	Operator-dependent, artifacts	Systematic reviews/meta-analyses; mostly observational (Level II–III)
Metastases [42,43]	Detect residuals, guide margins	↑ GTR, fast, low cost	Limited sensitivity small lesions	Retrospective series; small prospective cohorts (Level III → II)
Meningiomas [46]	CEUS for vascularity/margins	Safe dissection, less bleeding	Shadowing in calcified tumors	Observational series; technical notes (Level III)
Pituitary adenomas [49,50,51,52,53,54,55]	Identify MRI-negative microadenomas	Targeted resection	Limited field of view	Observational series; small systematic series (Level III)
Spinal tumors [56,57,58,59,60]	Myelotomy, cleavage plane, decompression	Safer resection	Inconsistent long-term outcomes	Prospective/retrospective cohorts; technical reports (Level II–III)
Degenerative (OPLL, DCM) [18,63,64,65,66]	Assess decompression, prognosis	Prevents undertreatment	Shadowing, subjective grading	Prospective/retrospective cohorts; exploratory prognostic data (Level II–III)
CSF webs/cysts [69,70,71,72]	Visualize webs, confirm CSF flow	Diagnosis when MRI inconclusive	Few large studies	Case series/reports (Level III–IV)
Trauma [76,77,78,79,80,81,82]	Intraoperatively confirm decompression	Immediate intraoperative feedback	No randomized trials	Retrospective cohorts; case series (Level III)

**Table 3:** Stepwise guide to setup, baseline sweep, intraoperative checkpoints, final verification and common artifacts/pitfalls with fixes. Parameters are starting points and should be adapted to anatomy and device, following ALARA (As Low As Reasonably Achievable); perform the final sweep after hemostasis with warm, de-gassed saline and before definitive hemostatic placement. DREZ: dorsal root entry zone; EOR: extent of resection; ALARA: As Low As Reasonably Achievable.

Scenario	Setup	Baseline (First Step)	Intraoperative Decision Point(s)	Final Check (Before Closure)	Artifacts/Pitfalls and Fixes
Glioma	Micro-convex 3–8 MHz plus linear 7–12 MHz; harmonics ON; start with wide depth then reduce; focus at deepest margin; warm de-gassed saline ready.	After dural opening: panoramic sweep; annotate margins/eloquent borders/deep limits; save reference images.	At ~50–70% EOR: refill, 360° oblique sweeps; redirect trajectory; reset gain/focus to the new target depth.	After hemostasis: slight overfill, evacuate microbubbles; remove/relocate hemostatics; comprehensive corner sweeps; save images/clips.	Air: irrigate + slight probe tilt. Blood: gentle suction, irrigation, lower gain. Hemostatic agents: can mimic residual—scan before placement or remove temporarily and re-scan. Bone edge shadow: change window/angle.
Intramedullary tumor	Linear 7–15 MHz (minimal pressure), Doppler ready; micro-convex 5–8 MHz if access constrained.	Pre-myelotomy: identify median sulcus vs. DREZ; map polar/feeding vessels; define tumor–cord plane.	After internal debulking: reassess cleavage plane; search for satellite nodules or syrinx connection; adjust depth/focus.	Confirm cord re-expansion and pulsatility; document absence of focal residual; minimize manipulation during final sweep.	Probe pressure on cord: reduce pressure. Dropout from surface blood: irrigate and pause. Window loss: enlarge dural opening slightly if safe.
Chiari/CSF disorders	Micro-convex 3–8 MHz; harmonics ON; Doppler for flow; conservative output (ALARA).	Baseline: assess CSF pulsatility at foramen magnum and tonsillar impaction; save reference.	After bony decompression: reassess flow; if limited, plan duraplasty/arachnoid lysis; repeat after each step.	After duraplasty/lysis: confirm restored pulsatility and absence of obstructive membranes; save images before closure.	False-negative flow from ventilation/positioning: coordinate with anesthesia. Air reverberation: fill dead spaces with saline. Patch interface: interpret against baseline.

## Data Availability

No new data were created or analyzed in this study. Data sharing is not applicable to this article.

## Abbreviations

The following abbreviations are used in this manuscript:

IOUS	Intraoperative ultrasound
MRI	Magnetic resonance imaging
CT	Computed tomography
OPLL	Ossification of the posterior longitudinal ligament
EOR	Extent of resection
GTR	Gross total resection
3D	Three-dimensional
CSF	Cerebrospinal fluid
SCI	Spinal cord injury
PSO	Pedicle subtraction osteotomy
CEUS	Contrast-enhanced ultrasound
DCM	Degenerative cervical myelopathy
MFI/SMI	Microflow or superb microvascular imaging
SWE	Shear-wave elastography
